# The Effect of Resection Angle on Stress Distribution after Root-End Surgery

**DOI:** 10.22037/iej.v13i2.19089

**Published:** 2018

**Authors:** Jaiane Bandoli Monteiro, Amanda Maria de Oliveira Dal Piva, João Paulo Mendes Tribst, Alexandre Luiz Souto Borges, Rubens Nisie Tango

**Affiliations:** a *Department of Dental Materials and Prosthodontics, São Paulo State University (Unesp), Institute of Science and Technology, São José dos Campos, SP, Brazil*

**Keywords:** Apicectomy, Cut Angle, Dental Stress Analysis, Endodontics, Finite Element Analysis, Resection Angle

## Abstract

**Introduction:**

This study aimed to investigate the influence of the resection angle on the stress distribution of retrograde endodontic treated maxillary incisors under oblique-load application.

**Methods and Materials:**

A maxillary central incisor which was endodontically treated and restored with a fiber glass post was obtained in a 3-dimensional numerical model and distributed into three groups according to type of resection: control; restored with fiber post without retrograde obturation, R45 and R90 with 45º and 90º resection from tooth axial axis, respectively and restored with Fuji II LC (GC America). The numerical models received a 45^º^ occlusal load of 200 N/cm^2^ on the middle of lingual surface. All materials and structures were considered linear elastic, homogeneous and isotropic. Numerical models were plotted and meshed with isoparametric elements, and the results were analyzed using maximum principal stress (MPS).

**Results:**

MPS showed greater stress values in the bone tissue for control group than the other groups. Groups with apicectomy showed acceptable stress distribution on the fiber post, cement layer and root dentin, presenting more improved values than control group.

**Conclusion:**

Apicectomy at 90^º^ promotes more homogeneity on stress distribution on the fiber post, cement layer and root dentin, which suggests less probability of failure. However, due to its facility and stress distribution also being better than control group, apicectomy at 45^°^ could be a good choice for clinicians.

## Introduction

Apical root resection is an endodontic microsurgery procedure [1] that removes damaged or infected tissue combined with the removal of the apical third of the root after failure in the endodontic treatment [2, 3] or when the removal of the intraradicular retainer is not indicated [4].

The damaged tissue in periapical lesions can be predictably regenerated after removal of intra-canal microorganisms through conventional endodontic treatment [5]. However, when the periodontal ligament is destroyed in the affected apical portion, the regeneration of this tissue may be unpredictable after surgery [6, 7] due to the immediate occupation of the apical root surfaces by epithelial cells present in bone tissue [8]. Anterior teeth usually show estimated success rate above 85% after endodontic microsurgery [5-7, 9].

Access to the root apex, the visibility of the lesion and the facility of apical sealing [4] are relevant criteria in choosing the resection angle [10]. In cases where the retrograde filling is not necessary, apicectomy in the oblique cutting plane is easier to perform and provides better accessibility than a perpendicular cutting plane. 

Considering biomechanical and biological aspects [11], it is preferable to use a perpendicular plane during apical resection [12]. Teeth that have their apex resected with specific angle showed apical bone rarefaction, and so it is better to avoid any presence of angles and chamfer at the apical portion level where the concentration of forces is able to trigger osteolysis caused by unfavorable concentration of tensile stress [13].

It is reported that the apical cutting angle can modify the isochromatic fringe patterns produced by the mechanical constraints in studies with photoelasticity [12]. An apical resection can modify stress distribution and tooth mobility [2, 12], so biomechanical studies should precede microsurgery [2]. 

Thus, the purpose of this study was to assess the biomechanical effect of apical root resection in periapical microsurgery varying the resection angle by three-dimensional finite element analysis (FEA). The hypothesis of this study was that different apical root angles do not interfere with the biomechanical behavior of a single-rooted tooth. 

## Materials and Methods

***Finite Element Analysis***

**Figure 1:**
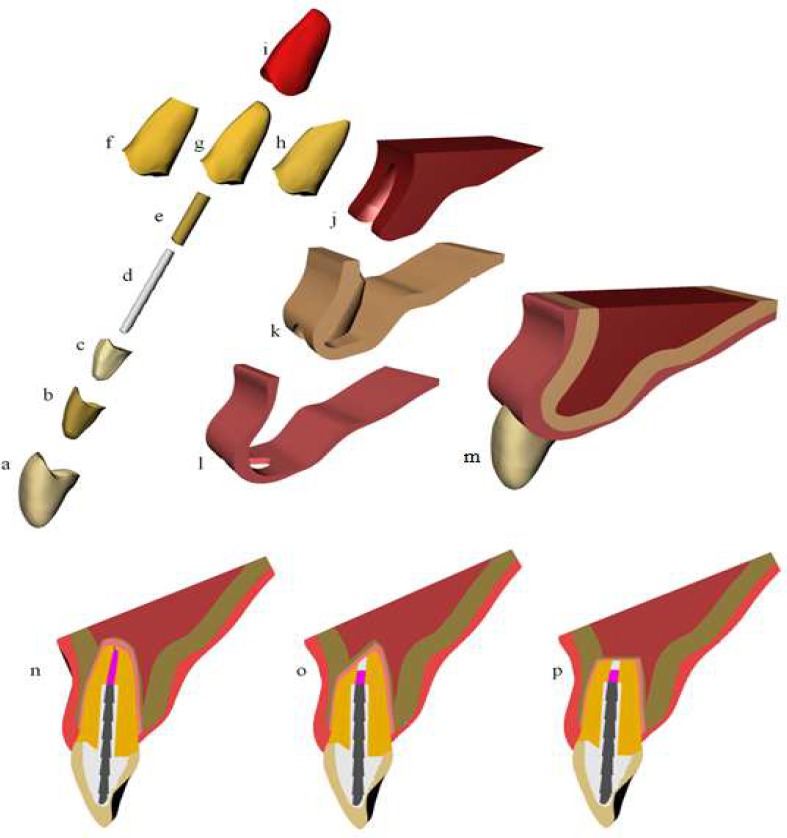
Schematic illustration of the sequentially performed procedures. *A)* Ceramic crown; *B)* Resin cement; *C)* Core; *D)* Fiber post; *E)* Resin cement; *F)* Root with the apical third resected at 90°; *G)* Root without resection; *H)* Root with the apical third resected at 45^°^; *I)* Periodontal ligament; J) Medullar bone; *K)* Cortical bone; *L)* Gums; *M)* Final geometry; *N)* Lateral vision of a incisor restored with a fiber post; *O)* Lateral vision of a incisor restored with a fiber post and with a resection at 45^°^; *P)* Lateral vision of a incisor restored with a fiber post and with a resection at 90^°^

A 3-dimensional geometric model previously validated was elected to perform this study [13]. A schematic illustration of the sequentially performed procedures is presented in Figure 1. The intact human maxillary central incisor was selected from São Paulo State University database (UNESP – ICT São José dos Campos). The total length of the tooth was 22 mm, with 9 mm of crown length and 13 mm of root length with the last 4 mm filled with gutta-percha. The alveolar bone crest was located 1 mm below the cementoenamel junction, supporting 12 mm of the root. The periodontal ligament was designed with 0.30 mm thickness. 

The numerical models were exported to computer-assisted design software (Rhinoceros 3D 5.0; McNeel 2010, North America, Seattle, WA, USA) and restored with a fiber glass post. The cement layer was created occupying the entire space between the post and the root dentin with 0.3 mm thickness [13, 14]. This model was kept as the control, and two more models were developed following the inter-treatment factors on the biomechanical response of the tooth, thereby simulating different resection angles including 45^º^ and 90^º^ [12].

The root resection was simulated in the two surgically treated models. For this, were performed a 3-mm apical root resection without a bevel angle (in 45-degree for one group and in 90-degree for another one), retro-preparation (cylinder shaped cavity with a 1.5-mm diameter and 3-mm depth), and MTA retrofilling on the surgically treated models. Both groups (45^°^ and 90^°^) simulated the complete resolution of the periapical bone lesion after surgical intervention, with recovery of the inner cortical bone (0.5-mm thick) and periodontal ligament layer [2].

During preprocessing, the models were exported in STEP files to computer aided engineering software (ANSYS 17.2, ANSYS Inc., Houston, TX, USA). The meshing of each structure was performed with solid quadratic tetrahedral elements with 10 nodes. Boundary conditions were defined with a 200 N force applied to a 5×1 mm area in the middle third of the palatal surface of the crown at a 45-degree angle. This area corresponded to the surface of the occlusal contacts [2, 15, 16].

A nodal displacement constraint was applied at the bottom and lateral surfaces of the cortical bone on the X, Y, and Z axes. All contacts were considered ideal. A linear structural analysis was performed, and all materials were considered linear, isotropic, and homogeneous (Table 1) [16-21].

The stress distributions in the restored teeth were analyzed using the Maximum Principal Stress criteria. 

**Table 1:** Distribution of mechanical properties of the materials

**Structure** **/material**	**Elastic modulus (GPa)**	**Poisson ratio**
Ligament	0.069	0.45
Dentin	18.6	0.32
Gums	0.003	0.45
Cortical Bone	13.7	0.30
Trabecular Bone	1.37	0.30
Rely X ARC cement (3M ESPE)	5.1	0.27
Gutta Percha	0.69	0.45
Fiberglass Post	49	0.28
Lithium Dissilicate IPS e.max Press (Ivoclar Vivadent)	82.3	0.22
Resin-modified glass-ionomer cement Fuji II LC (GC America)	10.8	0.30

**Table 2:** Group distribution according to the resection angle and numbers of nodes and tetrahedral solid elements

**Groups**	**Resection type**	**Nodes**	**Elements**
**Control**	-	262144	13294
**R45**	45^°^	231702	12987
**R90**	90^°^	228626	125659

## Results

**Figure 2:**
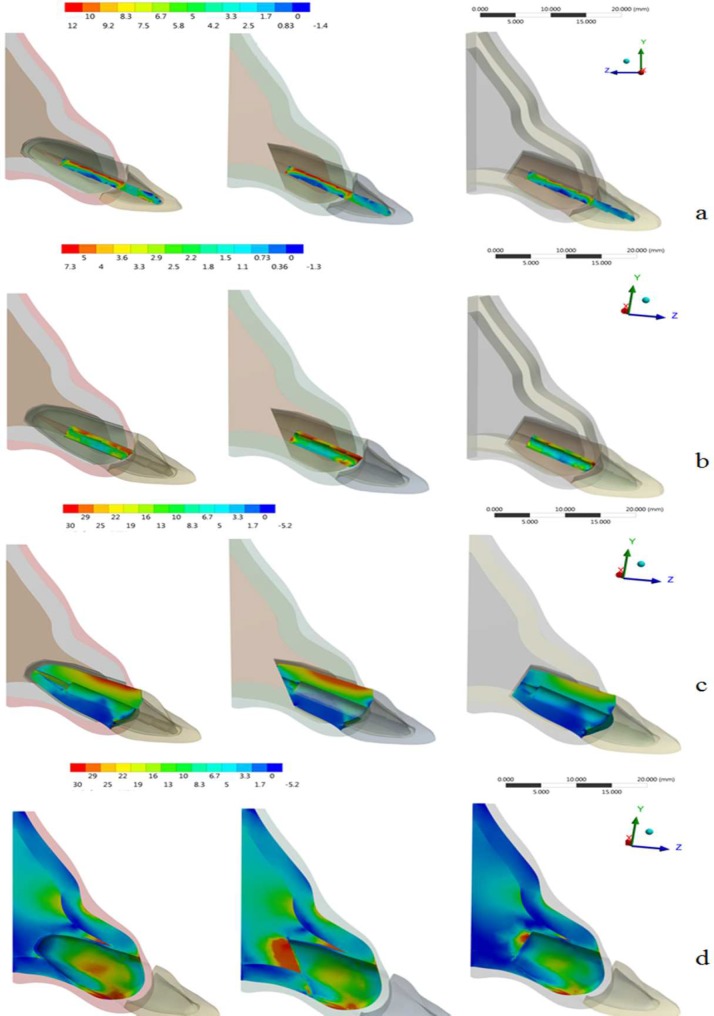
Maximum Principal Stress (MPa) distribution according to the structures. *A)* Fiber post; *B)* Cement line; *C)* root dentin; *D)* Bone tissue. From left to right: control group, R45 and R90

The results were analyzed using the Maximum Principal Stress (MPS) criteria (Table 2). Figure 2 shows the stress distribution in the numerical model for the control group and groups with 45^º^ and 90^º^ resection angles. 

In analyzing the energy dissipation coherence, the system displacement tendency occurs in the intra-alveolar direction with a deformation peak present in the incisal region, decreasing the rotation fulcrum after apicectomy with resection of 45^º^ and 90^º^.

***Stress distribution in fiber post***

Apicectomy procedure at different angles did not increase the stress peak generated inside the fiber post. The stress concentration occurred on buccal region of the post for all groups, however, with less magnitude for the R90 group (Figure 2A).

***Stress distribution on cement layer***

**Figure 3:**
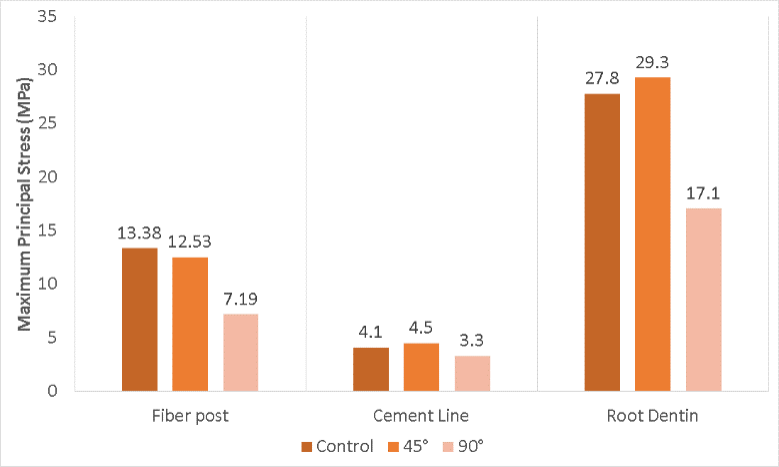
Higher values of Maximum Principal Stress (MPa) according to the structures (Fiber post, Cement line and Root dentin) and resection angle

The cement line in the R90 group presents better mechanical behavior when compared to the control and R45 groups (Figure 2B), where a slight stress concentration is observed in the cervical region. R45 group had a higher tensile peak stress on the cement layer than the control and R90 groups (Figure 3).

***Stress distribution on root dentin***

For control group, the stress generated on the root was concentrated along the buccal surface which increased in R45 group and decreased in R90 group (Figure 2C). The model with an apicoectomy at 45^º^ presented a higher tensile peak stress (Figure 3).

***Stress distribution on bone tissue***

The results show that the stress in bone tissue in the apical region was better distributed for the control group than the experimental groups. The presence of apical resection increased the stress concentration in the bone beside the apex, with higher intensity in group R45 (Figure 2D). 

## Discussion

Endodontic microsurgery is a resource used in cases of persistent or refractory periradicular pathology that does not heal after non-surgical retreatment [22] and is based on the use of equipment, instruments and materials that combine biological concepts with a practiced clinical outcome due to the production of predictable results in healing lesions of endodontic origin [23]. Therefore, different biomechanical responses can be observed when microsurgery is performed to remove the lesion associated with apical resection of a tooth with periodontal support [24]. In this study, the behavior of tooth models with intraradicular fiberglass post, total ceramic crown and surrounding alveolar bone structures in combination with representative clinical situations (total bone formation) were simulated after endodontic microsurgery with an apical resection at 90^º^ and 45^º^ angles. As a result, these events affected the biomechanical response of a tooth, rejecting the null hypothesis.

The success of parendodontic microsurgical treatment depends on determining factors that involve the apical anatomy of the teeth and surrounding tissues, the absence of periodontal involvement [25], surgical techniques with apical root resection, as well as the use of biocompatible materials in the apical retro filling [23, 25, 26]. In this study, finite element analysis (FEA) models were constructed with resection of the apical portion at 45^º^ and 90^º^ in relation to the long axis of the root. This resection is done to optimize elimination of apical branches, to maintain a limited root length, to reduce the number of open dentinal tubules, and bacterial infiltration [27].

However, it is important to note that apical resection can lead to exposure of the dentinal tubules and cause risk of bacterial contamination [28, 29]. Therefore, root end preparation is often advocated after the basic steps of endodontic surgery and a suitable root end filling material is inserted [29]. Retrofilling at a preparation depth of at least 3 mm is done to provide a safe and effective apical seal [8, 26]. Glass ionomer cement was selected in this work due to lower bacterial microleakage after one year of follow-up [30].

The restoration of upper anterior teeth presents a great challenge in everyday clinical practice [16], and despite the development of materials and techniques, intraradicular posts may interfere in the mechanical resistance of an endodontically treated tooth, increasing the risk of damage to the remaining structure [31]. However, factors such as coronal tissue preservation and the use of glass fiber posts with similar elastic properties to dentin and effective adhesion to resin cement are necessary to obtain the expected clinical success [32]. This material presents lower intraradicular stress because it induces a stress field quite similar to a natural tooth, except for the cervical portion stress concentration [33]. Regarding the results for the post structure, the resection angle was significant due to the fact that R90 group better distributed the stress, while R45 showed stress values similar to control group. 

Stress caused by occlusal forces may occur where the maximum stress exists in the weakest point, which is often related to the cementation line in endodontically treated and restored teeth with intraradicular posts [34]. The biomechanical behavior for cement line was similar to fiber post. In the models without resection and with 45^º^ angle resection, a slight stress concentration could be observed in the initial part of the third cervical portion of the cement line, while better stress distribution occurred in the model with 90^º^ resection. Tested models demonstrated higher (R45) and lower (R90) peak stress values than control group, suggesting higher and less probability of adhesive failures [35].

In the model without resection, maximum stress concentration of the root dentin, was mainly observed in the palatal portion of the root. This behavior was also observed in previous studies that numerically simulated the stress distribution in teeth endodontically restored with intraradicular posts [13, 21, 36]. In the presence of coronary remnants and intraradicular post, the stress is redistributed in the outer superficial regions of the root [13, 37]. A recent study [13] affirms that tensile stress in dentin is proportional to increased bone loss. The present study can suggest that root resection at 45 degrees is another factor to increase the tensile stress, and that 90 degree resection can improve stress distribution in situations where root resection is needed. This behavior can be explained by the larger surface area of the dental structure to receive the tensile stress[38].

The presence of normal periodontal tissue is a significant factor for tooth support, even when the apical resection is done with up to 6 mm of root length; therefore, adequate periapical healing is essential not only for endodontic success, but also for biomechanical re-establishment [2, 3, 39]. Considering bone tissue, all groups showed stress concentration on the cervical third of the buccal face, decreasing for R45 and R90 groups, respectively. Also, resected models showed stress concentration on the resected area, suggesting that angles concentrate more energy. Resecting the root at 45 degrees can promote higher damage to bone tissue; however, both experimental conditions showed micro-strain values inferior to the physiological limit, which suggests no bone resorption [40]. In this way, R90 is the better suggested protocol.

FEA is a numerical method that helps to solve the difficult problems in the different sciences, per example, clinical situations regarding the difficulty of reproducing all variations of clinical situations, different types of teeth and morphology, periodontal condition, endodontic procedures and restored areas. This analysis accompanies several assumptions considered to be simpler and faster: the evaluated structures were considered homogeneous, isotropic and linear, as well as having a perfect boundary condition [24]. One of this study’s limitations would be that the apical resection condition was only simulated in two distinct forms using 45^º^ and 90^º^. Other types of backfill fillers such as MTA, amalgam or other resin cements were not simulated. In order to avoid increasing the number and complexity of the models, only these two angles and a filling material were reproduced and compared with periapical microsurgery in the absence of apicoectomy. However, further studies with improved models are needed to more accurately predict the biomechanical responses of the anterior tooth after periapical microsurgery and surrounding alveolar bone structures. 

## Conclusion

Within the limitations of this finite element analysis, it is possible to ascertain that apicectomy at 90^º^ promotes more homogeneity on stress distribution on the fiber post, cement layer, root dentin and bone tissue, which suggests less probability of failure.
